# Legionella Pneumonia Undetected by Repeated Urinary Antigen Testing With Ribotest® Legionella

**DOI:** 10.7759/cureus.74035

**Published:** 2024-11-19

**Authors:** Yasushi Murakami, Mika Morosawa, Yasuhiro Nozaki, Yoshio Takesue

**Affiliations:** 1 Department of Respiratory Medicine, Tokoname City Hospital, Tokoname, JPN; 2 Department of Clinical Infectious Diseases, Tokoname City Hospital, Tokoname, JPN

**Keywords:** bronchoscopy, legionella pneumonia, loop-mediated isothermal amplification, ribotest® legionella, urinary antigen test

## Abstract

This report presents a patient with *Legionella* pneumonia (LP), initially presented with fever and mild hypoxemia, with subsequent progression to severe pneumonia during hospitalization. Despite multiple negative urinary antigen tests using Ribotest^®^ *Legionella*, the diagnosis was confirmed via the loop-mediated isothermal amplification method of lower respiratory tract secretions. This case highlights the diagnostic limitations of Ribotest^®^ *Legionella* and emphasizes the importance of a comprehensive diagnostic strategy, incorporating nucleic acid amplification tests or culture in suspected patients with LP. Early recognition of these diagnostic challenges is critical for optimizing patient outcomes.

## Introduction

*Legionella* pneumonia (LP) is a severe type of pneumonia primarily caused by *Legionella pneumophila *and is associated with increased morbidity and mortality. LP requires distinct antibiotic regimens compared to other forms of pneumonia caused by typical respiratory pathogens, and delays in initiating appropriate therapy can result in adverse patient outcomes [1]. Prompt recognition of *Legionella *infection is critical in the management of pneumonia, particularly in patients with severe illness or those at high risk.

The urinary antigen test (UAT) is a widely used initial diagnostic test for LP owing to its simplicity, rapid turnaround time, and relative cost-effectiveness [2-4]. However, conventional UATs are designed to detect lipopolysaccharide (LPS) in *Legionella pneumophila *serogroup 1 (SG1). *L. pneumophila *SG1 was the predominant causative pathogen of LP, accounting for 79.3% of cases, followed by non-SG1 *L. pneumophila* (11.4%), *Legionella bozemanae *(3.6%), *Legionella dumoffii *(3.6%), *Legionella micdadei *(1.4%), and *Legionella longbeachae *(0.7%) [5]. A systematic review of the diagnostic accuracy of conventional UATs for legionellosis reported an overall sensitivity of 0.79, improving to 0.86 when limited to *L. pneumophila *SG1 [3]. Consequently, a significant proportion of patients with LP remain undiagnosed with conventional UATs, highlighting the need for novel point-of-care tests (POCTs) that can detect a broader range of *Legionella *species and serogroups.

The Ribotest^®^ *Legionella *(Asahi Kasei Pharma Corporation, Tokyo, Japan) is a newly developed UAT designed to detect all serotypes of *L. pneumophila*, aiming to overcome the limitations of conventional UATs [6,7]. However, its effectiveness in clinical practice remains insufficiently validated, with limited reports on diagnostic errors. Herein, we present a patient with LP diagnosed via loop-mediated isothermal amplification (LAMP) of lower respiratory tract secretions, despite multiple negative results of the Ribotest^®^ *Legionella*.

## Case presentation

A 55-year-old male presented to the emergency department with a two-day history of persistent fever, generalized fatigue, and shortness of breath. His medical history included hypertension, and he had no history of immunocompromised disorders. He was a current smoker with a 25 pack-year history. He had no recent travel history or exposure to hot springs, public baths, or soil contact. On admission, the patient was alert with a temperature of 40.9 °C, respiratory rate of 27 breaths/min, blood pressure of 159/79 mmHg, heart rate of 111 bpm, and oxygen saturation of 92% on ambient air.

**Figure 1:**
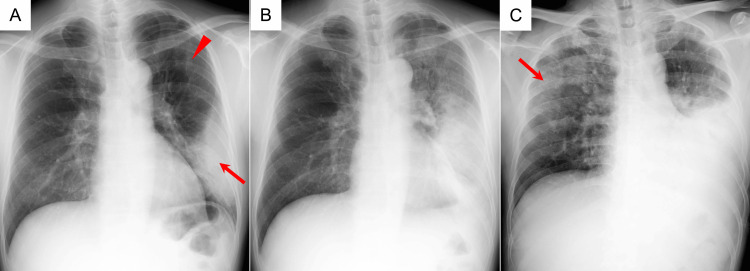
Chest X-ray findings during hospitalization (A, B, C) Chest X-ray on day 0 (admission), day 2, and day 6, respectively. (A) Wedge-shaped infiltration in the left lower lobe adjacent to the pleura (arrow), along with GGOs in the left upper lobe (arrowhead). (B) The consolidation and GGOs expand to involve the entire left lung. (C) Collapse of the left lower lobe and extension of GGOs into the right lung (arrow). GGOs, ground-glass opacities

**Figure 2:**
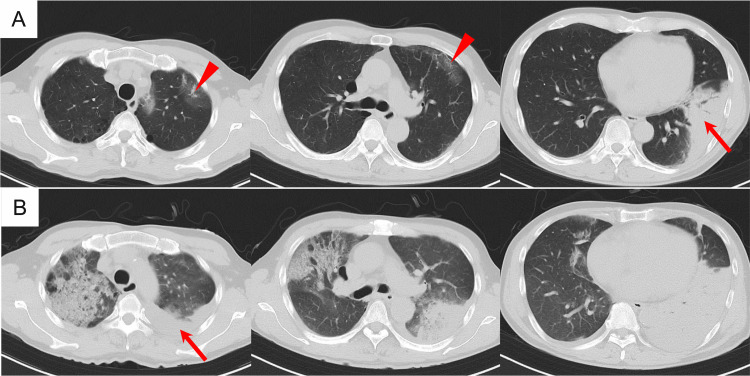
Chest CT during hospitalization (A, B) Chest CT on day 0 (admission) and day 5, respectively. (A) Wedge-shaped consolidation with an air bronchogram in the left lower lobe adjacent to the pleura (arrow), along with scattered GGOs in the left upper lobe (arrowheads). (B) Complete collapse of the left lower lobe, with a small pleural effusion (arrow). A new infiltrate is observed in the left upper lobe, and GGOs have extended into the right upper lobe. GGOs, ground-glass opacities

The blood test results obtained at admission are shown in Table 1. Elevated white blood cell count (15,800/mm³) and CRP level (29.8 mg/dL) were observed. The albumin level was mildly reduced (3.3 g/dL), and the aspartate aminotransferase level was slightly elevated (40 IU/L). Additionally, hyponatremia (129 mmol/L) and hypokalemia (3.1 mmol/L) were noted. Arterial blood gas analysis was not performed. Chest radiography and CT revealed a wedge-shaped consolidation with an air bronchogram in the left lower lobe, along with scattered ground-glass opacities (GGOs) in the left upper lobe (Figure 1A, Figure 2A). Mild emphysema was also observed in both lungs. A polymerase chain reaction test for SARS-CoV-2 via pharyngeal swab was negative. Similarly, the UAT using Ribotest^®^ *Legionella *provided a negative result. Blood and sputum samples were sent for culture.

**Table 1:** Laboratory test results on admission Alb, albumin; ALT, alanine aminotransferase; AST, aspartate aminotransferase; BUN, blood urea nitrogen; CK, creatine kinase; Cr, creatinine; Hgb, hemoglobin; LDH, lactate dehydrogenase; PLT, platelets; T-Bil, total bilirubin; TP, total protein

Parameter	Result		Normal range
WBC	15.8	×10^3^/mm^3^	4.0-9.0
Neutrophils	93.5	%	40.0-60.0
Lymphocytes	3.5	%	20.0-40.0
RBC	4.3	×10^6^/mm^3^	4.0-5.5
Hgb	14	g/dL	13.0-17.0
PLT	210	×10^3^/mm^3^	150-350
TP	6.5	g/dL	6.6-8.1
Alb	3.3	g/dL	4.1-5.1
AST	40	IU/L	13-30
ALT	29	IU/L	10-42
LDH	223	IU/L	135-225
CK	96	IU/L	59-248
T-Bil	0.6	mg/dL	0.2-1.2
BUN	16.6	mg/dL	8.0-20.0
Cr	0.91	mg/dL	0.65-1.07
Sodium	129	mmol/L	138-146
Potassium	3.1	mmol/L	3.6-4.9
Chloride	91	mmol/L	99-109
CRP	29.8	mg/dL	0-0.3

**Figure 3:**
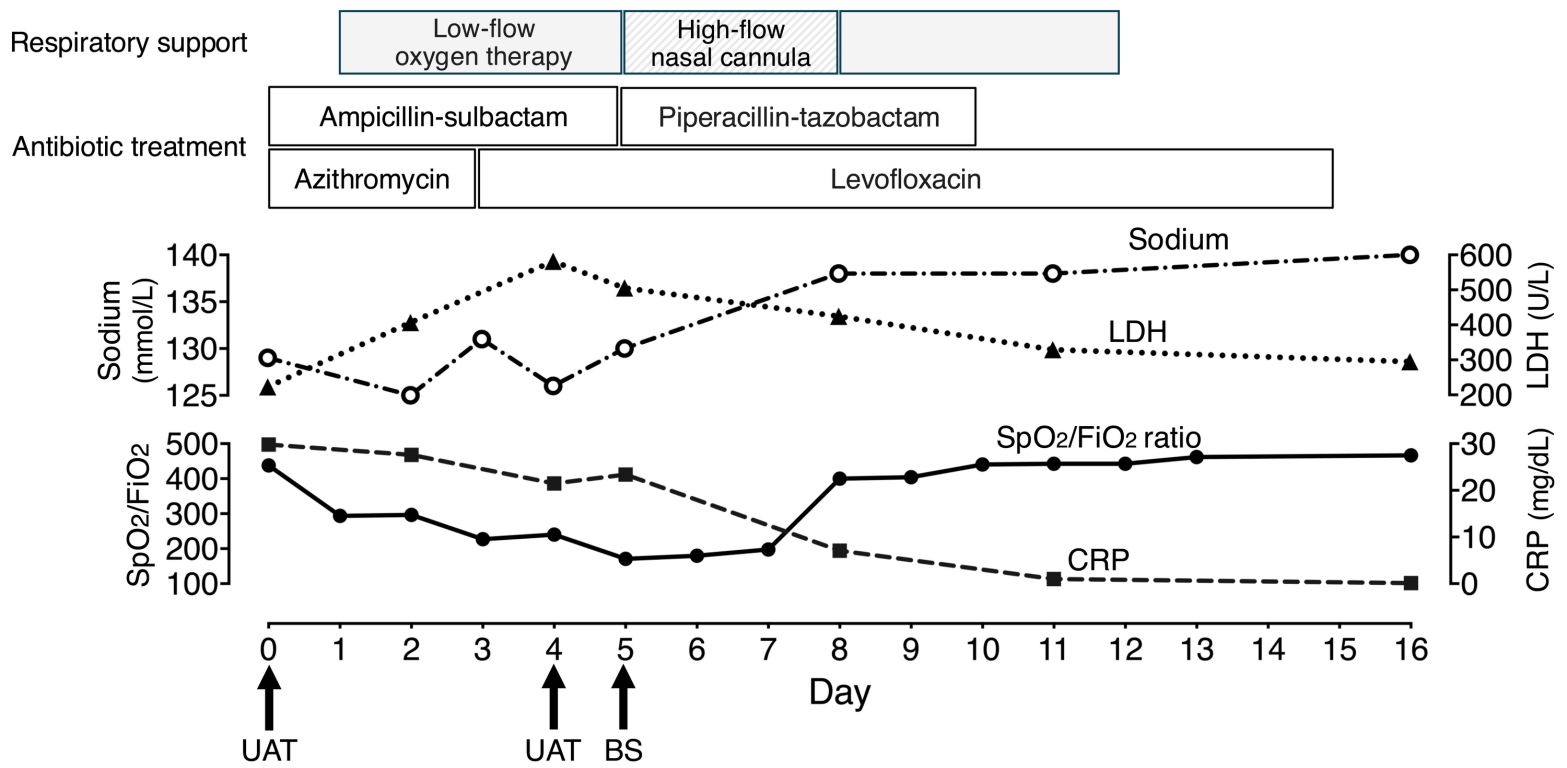
Clinical course during hospitalization Day 0 represents the day of admission. BS, bronchoscopy; FiO2, fraction of inspired oxygen; LDH, lactate dehydrogenase; SpO2, pulse oximetry saturation; UAT, urinary antigen test

The clinical course of hospitalization is described in Figure 3. The patient was diagnosed with community-acquired pneumonia (CAP) and treated with intravenous ampicillin-sulbactam (12 g/day) and azithromycin (500 mg/day). Blood cultures were negative for any organisms. Sputum examination detected two types of gram-negative bacteria (*Escherichia coli *and *Haemophilus influenzae*) both susceptible to the initial antibiotic regimen. However, the patient’s high fever persisted, and his respiratory condition progressively worsened. In addition to persistent inflammation, laboratory tests revealed progressive hyponatremia and an elevated lactate dehydrogenase (LDH) level. Imaging studies revealed the progression of pulmonary infiltrates in both lungs (Figure 1, Figure 2).

Suspecting LP, azithromycin was replaced by intravenous levofloxacin (500 mg/day) on day 3 after admission. However, the patient’s respiratory condition did not improve. A second Ribotest^® ^*Legionella *UAT, performed on day 4, was negative. On day 5, a bronchoscopic examination was performed under high-flow nasal oxygen therapy, revealing diffuse edematous changes in the lower airway mucosa, and orange secretions were collected for routine bacterial culture and *Legionella *testing, including LAMP assay and culture. Ampicillin-sulbactam was replaced by intravenous piperacillin-tazobactam (18 g/day), and levofloxacin was continued. The patient’s respiratory condition gradually improved, and he was discharged on day 16. Although culture tests from the bronchoscopy-obtained specimens were negative, the LAMP assay was positive, confirming a diagnosis of LP.

## Discussion

This report presents a case of LP that could not be diagnosed through repeated UAT using Ribotest^®^ *Legionella*. This case highlights two key points in the management of LP: (1) the novel UAT kit may fail to detect LP in patients with progressive respiratory failure even with serial testing, and (2) when epidemiological and/or clinical factors suggest LP, it is important to consider more sensitive diagnostic methods than UATs, such as nucleic acid amplification tests (NAATs) or culture.

*Legionella* are gram-negative bacilli commonly found in environmental water sources and are a frequent cause of severe CAP, with a mortality rate of 5-10% [1]. As *Legionella* replicates inside alveolar macrophages, it is resistant to β-lactam antibiotics, the mainstay of first-line treatment for CAP. However, it is susceptible to agents with high intracellular penetration, such as quinolones or macrolides [1]. Although available evidence is limited, bacterial coinfection is relatively common in patients with LP, particularly *Streptococcus pneumoniae *coinfection [8]. In the present case, the detection of *E. coli *and *H. influenzae *in the sputum culture test suggests the possibility of coinfection.

Delays in initiating antibiotics targeting *Legionella *may lead to worse patient outcomes [1]. Prompt diagnosis and timely treatment are essential in LP management. Ribotest^®^ *Legionella*, developed in 2019, is a novel UAT kit that utilizes immunochromatography to detect *L. pneumophila *ribosomal protein L7/L12, in addition to conventional *L. pneumophila *SG1 LPS. This novel assay has demonstrated diagnostic accuracy comparable to that of conventional UATs and has the potential to detect non-*L. pneumophila *species and all *L. pneumophila *serotypes, making it a promising POCT for pneumonia management [6,7]. However, evidence on its diagnostic accuracy is limited, and further large-scale studies are needed to determine its effectiveness in clinical practice. In this case, *Legionella *infection was not detected using Ribotest^®^ *Legionella,* despite repeated testing, and the final diagnosis was made using the LAMP method on bronchoscopy-obtained lower respiratory tract samples.

Several factors could explain the multiple negative UAT results. First, the early stage of infection and relatively low disease severity at the time of testing. After the onset of LP, urinary antigen levels increase as the bacterial load in the lungs increases over time [9-11]. Therefore, repeated testing can be helpful when the initial UAT result is negative. Notably, urinary antigen levels may increase to a detectable level approximately three days after symptom onset [9,10]. Motokura et al. reported a patient with LP caused by *L. pneumophila *SG1, where the second Ribotest^®^ *Legionella* UAT had successfully diagnosed the infection four days after an initial failure of diagnosis upon admission [11]. Additionally, UAT sensitivity was directly correlated with the severity of LP, with higher sensitivity in more severe cases [12]. In the present case, despite four days since the onset and the progression of respiratory failure, the second UAT failed to detect *Legionella* infection. Second, a previous study suggested that antigens are released into urine intermittently in some cases of LP [4], which may explain the negative UAT results. Third, the infection may have been caused by a *Legionella *species other than* L. pneumophila*, accounting for approximately 9.3% of LP in Japan [5]. Considering the repeated UAT failures, we believe that this is the most likely explanation for false negative results with Ribotest^®^ *Legionella*. Oda et al. reported a case of* L. longbeachae *pneumonia that Ribotest^®^ *Legionella *failed to diagnose [13]. Similarly, Shinomiya et al. described a case of *L. longbeachae *pneumonia, where the first UAT was negative, but the second test identified the infection [14]. A high antigen load is required to detect non-*L. pneumophila *species using the Ribotest^®^ *Legionella*, leading to lower sensitivity compared to detecting *L. pneumophila *[6,14].

Our case highlights the importance of advanced diagnostic tests, including NAAT and culture, and emphasizes the need for physicians to incorporate these tests to improve diagnostic accuracy even when Ribotest^®^ *Legionella* is available. While advanced tests, including the LAMP, are more sensitive than the UAT and capable of detecting all *Legionella *species, they are more expensive and difficult to perform in many facilities [4,15]. Therefore, it is appropriate to reserve these tests for patients with a high suspicion of LP and negative UAT results. Recent international guideline for CAP recommends *Legionella* UAT, and/or additional tests, for patients with known epidemiological risk factors and/or severe pneumonia [2]. However, these risk factors are insufficient to predict LP, as most cases are sporadic [16], and patients may initially present with mild symptoms, even if they later require admission to the intensive care unit [17]. In this case, the patient had no history of travel or exposure to a local *Legionella *outbreak and presented with mild hypoxemia on admission. A more practical approach to predicting LP would involve the patient’s symptoms, vital signs, and diagnostic workup. For example, a diagnostic score consisting of six clinical and laboratory parameters (i.e., high fever, high CRP, high LDH, thrombocytopenia, hyponatremia, and unproductive cough) has demonstrated good diagnostic reliability in several studies [18]. Additionally, multi-lobar or multi-segmental lung consolidations with GGOs were well-documented CT features of LP [19]. Furthermore, orange-colored lower respiratory tract secretions may be a characteristic feature of LP [20]. These findings allowed us to pursue the diagnosis of LP, despite repeated negative UAT results and the detection of the other pathogens in the sputum specimens.

## Conclusions

Ribotest^®^ *Legionella* UAT kit may fail to diagnose LP, even with repeated testing. Physicians should recognize this limitation and carefully assess the possibility of *Legionella *infection in the management of pneumonia. In patients with high clinical suspicion of LP, it is crucial to follow a multimodal approach, including NAAT or cultures. Additionally, negative UAT results should not rule out LP. The development and implementation of rapid and highly sensitive diagnostic tools across various healthcare settings are needed to further improve patient outcomes.
